# The effect of *RORA* (RAR-Related Orphan Receptor Alpha) receptors on litter size in Akkaraman sheep breed

**DOI:** 10.22099/mbrc.2023.47336.1827

**Published:** 2023

**Authors:** Serbulent Yiğit, Selim Kul, Recai Aci, Adem Keskin, Tuğçe Tuygun, Esra Duman

**Affiliations:** 1 Department of Genetics, Faculty of Veterinary Medicine, Ondokuz Mayis University, Samsun, Turkiye; 2 Department of Medical Biology, Graduate Institute, Ondokuz Mayis University, Samsun, Turkiye; 3 Faculty of Veterinary Medicine, Department of Animal Breeding, Fırat University, Elazig, Turkiye; 4 Department of Biochemistry, Faculty of Veterinary Medicine, Ondokuz Mayıs University, Samsun, Turkiye; 5 University Faculty of Medicine, Department of Biochemistry, Aydın, Turkiye; 6 Department of Parasitology, Faculty of Veterinary Medicine, Ondokuz Mayıs University, Samsun, Turkiye; 7 Department of Gastrology and Hepatology, Faculty of Medicine, Kocaeli University, Kocaeli, Turkiye

**Keywords:** Sheep, RORA, Insertion/deletion (Indel), Mmutation, Litter size

## Abstract

In this study, the relationship between *RORA* 23bp indel genotype and allele frequency with twin pregnancy, fertility, live weight and milk yield in 106 female Akkaraman ewes raised in Elazığ province was investigated. In the study conducted in Elâzığ province, 10ml milk was collected from 106 Akkaraman sheep and DNA was extracted from these milk. In *RORA* 23bp indel genotype frequency, *DD* genotype was found more than *ID* and *II* genotypes and *RORA* 23bp indel ın allele frequency, the *D* allele was found to be higher than the I allele. In both the first and second parity, the twinning rate was found to be lower. In both the first and second parity, the twinning rate was higher in the *DD* genotype, and it was observed that this genotype promınated mıddle lıvestock weıght and mılk yıeld. According to the results of our study, mutations in the *RORA* gene, which is a gene affecting reproductive efficiency in sheep, do not have a positive effect on fertility and twinning rate in Akkaraman sheep. To sum, this study provided theoretical references for the comprehensively research of the function of *RORA* gene and the breeding of Akkaraman Sheep. The 23-bp indel variants can be considered as molecular markers for litter size of sheep for marker-assisted selection breeding.

## INTRODUCTION

According to FAO data, Turkey is the 7th country worldwide in terms of total sheep population, with around 31 million sheep. Almost half of the sheep population in Turkey, which is currently around 45%, is composed of a fat-tailed breed known as the Akkaraman [1]. This breed is specifically bred in the central Anatolia region and is capable of thriving in spite of poor climatic conditions and low-quality pasture. The Akkaraman breed is primarily utilized to meet the demand for mutton in Turkey. Studies have shown that the Akkaraman sheep breed exhibits a range of lamb birth weights, from 3.81 kg to 4.56 kg [2]. The breed also displays significant variations in terms of birth weight, indicating the need for studies aimed at enhancing lamb birth weights and growth characteristics. Such studies will be crucial in improving the overall productivity and growth potential of this breed.

Quantitative genetic studies conducted on sheep have revealed that certain genetic factors can impact lamb birth weight. The heritability of lamb birth weight in various sheep breeds has been reported to range between 0.15 and 0.24 [3]. As a result, the implementation of genomic selection is viewed as a potentially effective method for enhancing lamb birth weights.[4] Although it has a relatively small impact on livestock breeding, certain candidate genes have been identified that can help improve polygenic traits such as growth, and contribute to the precise estimation of genetic value in different livestock species, including sheep [5].

Nuclear receptors are soluble proteins that target gene transcription (genetic replication) but are located in the cell cytoplasm and/or nucleus, can bind to specialized DNA regulatory proteins and are precursor regulators of transcription. They affect DNA transcription in the cell nucleus by interacting with heat shock proteins (HSP) through their cytoplasmic receptors. They are classically defined as ligand-activated transcription factors [6].

ROR-alpha is generally expressed in many tissues and cell types, including organs critical for metabolic homeostasis, such as skeletal muscle, retina, spleen, testis, lung, liver, white and brown adipose tissue, but is most expressed in the brain. It is especially expressed in the cerebellum and thalamus [7]. Studies have shown that Retinoic acid receptor alpha (*RORA*) is a possible transcriptional target of both androgen and estrogen receptors [8]. *RORA* is involved in many different processes such as neuronal development, metabolism and immunity. Recent research has focused on the effects of RORA on various systems, including the reproductive system. RORA is thought to modulate androgen and estrogen production and therefore has a significant effect on the reproductive system [9]. Studies in both animals and humans show that RORA acts on reproductive functions through regulation of androgen and estrogen production.

Genomic mutation, called insertion/deletion (indel), occurs when nucleotide fragments of varying lengths are inserted or deleted in the same region of the genome between closely related species or between different individuals of the same species. Indels have been widely used in marker-assisted selection (MAS) breeding in recent years due to their frequency in eukaryotic genomes and easily identifiable features [10]. In a study using a Chinese Australian White sheep population (n=932), examination of different introns of the FecB gene revealed that only a 10 bp indel was significantly associated with ewe litter size, suggesting that the deletion/deletion (DD) genotype is detrimental for fertility. These findings will provide a new perspective towards developing a more robust basis for accelerating molecular reproduction in sheep through DNA markers in a marker-assisted selection strategy [11]. The study to determine the effect of DNMT3B on litter size in goats showed that the 3-bp indel in DNMT11B was significantly associated with firstborn litter size, which could be used for marker-assisted selection (MAS) for goat breeding [12]. In order to fully explore the functions of MARCH1 in goat reproduction, the mRNA expression of this gene was investigated in different tissues of goats, indicating that MARCH1 plays a crucial role in fertility and three novel indels in goat MARCH1 could be used as effective molecular markers for marker-assisted selection of goats for reproduction in the future [13]. Therefore, it is crucial to carry out additional studies to discover further significant indel mutations in candidate genes related to reproduction. Given the known function of the RORA gene, it is proposed that RORA has the potential to be a candidate gene that could impact the reproductive characteristics of animals. In this study, the relationship between *RORA* 23bp indel genotype and allele frequency with twin pregnancy, fertility, live weight and milk yield in 106 female Akkaraman ewes raised in Elazığ province was investigated.

## MATERIALS AND METHODS

We collected 10 ml of milk from 106 Akkaraman sheep in our Study Elâzığ city buildings. Table 1 contains the descriptive information of the sheep breed which used in the study.

**Table 1:** Descriptive information of 106 female Akkaraman sheep included in the study.

**Parameter**		**Female white sheep n:106**
**Mean age X±SD**		3.89±0.88
**Chest circumference (cm) X±SD**		105.65±6.03
**History of mastitis**	available n (%)	5 (4.7)
	none n (%)	101 (95.3)
**Disease history**	available n (%)	5 (4.7)
	none n (%)	101 (95.3)
**Live weight**	weak n (%)	15 (14.2)
	medium n (%)	67 (63.2)
	Heavy n (%)	24 (22.6)
**Milk yield**	weak n (%)	2 (1.9)
	medium n (%)	58 (54.7)
	Good n (%)	46 (43.4)
**2021 (1st parity) n (%)**	Single	80 (75.5)
	Twin	22 (20.8)
	Empty	4 (3.8)
**2022 (2nd parity) n (%)**	Single	95 (89.6)
	Twin	11 (10.4)
**Number of births n (%)**	One	4 (3.8)
	Two	34 (32.1)
	Three	42 (39.6)
	Four and above	26 (24.5)

We obtained DNA from these milks. We performed PCR using the Rora gene F:GGATGGGGCTG GTGGATTA and R:CAGGTGGTGAGCCATCTTGG primers from the DNAs we obtained. Our PCR conditions were as follows. 2 µl of genomic DNA (10 ng/ul), 1 ul of each primer (10 Um), 2.5 µl of PCR buffer (containing 1.5 mM MgCl_2_), 1 µl dNTPs (200 lM), and 1 µl DNA polymerase (1 units of Taq- Thermo Fisher Scientific ), and 17.5 µl ddH_2_O made up the entire reaction mixture (25 µl). The final step was to directly analyze 10 µl PCR results by electrophoresis on 3% agarose gels stained with ethidium bromide. Band size can be used to differentiate genotypes. PCR was performed using specific primers and agarose gel electrophoresis revealed that the 23-bp indel formed three genotypes: homozygous insertion genotype (insertion/insertion: II, 195 bp), deletion genotype (deletion/deletion: DD, 172 bp) and hetero-zygous genotype (insertion/deletion: ID, 195 bp and 172 bp).

SPSS for Windows 22.0 program was used for statistical analysis. Continuous variables obtained from the study were presented as mean ± standard deviation, categorical variables were presented as n (percentage frequency) and P values below 0.05 were considered statistically significant. Chi-square test was used to compare categorical variables.

**Figure 1:**
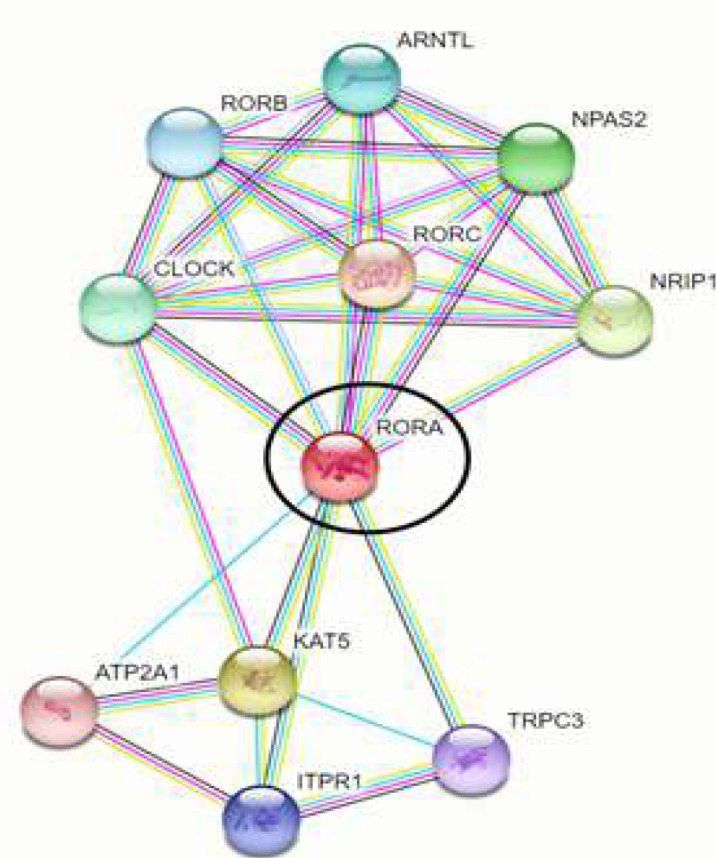
Interactions of RORA protein, according to STRING database predictions: RORA protein is evidenced with a ellipse.

STRING database annotates the functional interactions among the proteins in a cell. Analyzing the RORA protein with STRING database, predicted the functional partners of the protein with high confidence (score: 0.7) was found as follows: KAT5, ATP2A1, ITPR1, TRPC3, CLOCK, RORC, NRIP1, NPAS2, ARNTL, RORB (Fig. 1). 

## Results

The relationship between the number of offspring and genotype distribution in the first and second parity was evaluated in Table 2.

**Table 2:** Genotype distribution according to the number of offspring in the first and second parity

**Parity**	**Number** **of offspring**	**Genotype n (%)**			$x^{2}$	**df**	**P**
		**DD**	**ID**	**II**			
**Frist parity**	Single	44 (74.6)	27 (75.0)	9 (81.8)	1.813	4	0.770
	Twin	13 (22.0)	8 (22.2)	1 (9.1)			
	Empty	2 (3.4)	1 (2.8)	1 (9.1)			
**Second parity**	Single	54 (91.5)	33 (91.7)	8 (72.7)	3.768	2	0.152
	Twin	5 (8.5)	3 (8.3)	3 (27.3)			

There was no significant correlation between the number of offspring and genotype distribution in both the first and second parity. Genotype distribution according to milk yield and live weights of White Karaman ewes included in the study is presented in Table 3.

**Table 3:** Genotype distribution of Ak karaman ewes according to milk yield and live weight

**Parity**	**Number** **of offspring**	**Genotype n (%)**			$x^{2}$	**df**	**P**
		**DD**	**ID**	**II**			
**Live weight**	Weak	7 (11.9)	7 (19.4)	1 (9.1)	5.623	4	0.229
	Middle	34 (57.6)	25 (69.4)	8 (72.7)			
	Heavy	18 (30.5)	4 (11.1)	2 (18.2)			
**Milk yield**	Weak	1 (1.7)	1 (2.8)	0	1.476	4	0.831
	Middle	30 (50.8)	22 (61.1)	6 (54.5)			
	Good.	28 (47.5)	13 (36.1)	5 (45.5)			

There was no significant relationship between genotype distribution according to milk yield and body weight of Ak karaman ewes.

## Discussion

Akkaraman breed adult sheep have white bodies and possess a coarse, mixed fleece. It has high adaptaton to regional conditions. The tail is fat and large. The tail vertebrae make an S-bend at the tip. live weight can reach up to 45-50 kg. The wool yield is 1.5-2 kg, the length of the loops is 8-12 cm, the wool yield is 62-70%, the milk yield is 50-60 kg, the lactation period is 140-150 days, and the twin birth rate is around 20-30%. The development level of the lambs is medium and after weaning, they can reach 20-22 kg carcass weight after three months of fattening [14]. Fertility and body weight are complex traits that are influenced by multiple genes and environmental factors. It is widely recognized that conventional methods have limitations in improving these quantitative traits due to their inheritance patterns, which are expressed later in life, low heritability, and time-consuming process [15]. The genetic advancement of litter size and growth rate through conventional breeding methods usually ranges from 1 to 2%, similar to other quantitative traits [16]. Before making any decisions regarding genetic improvement of a breed, it is crucial to identify the genetic variability and major mutations in economically significant traits. Therefore, the aim of this study was to determine the genetic polymorphisms of vital traits such as twinning rate, live weight and milk yield in Akkaraman sheep breed. One of the criteria of economic value in sheep breeding is the high rate of fertility of ewes. For this purpose, many sheep breeding research papers have been published in which fertility rate is determined [17].

Nguyen et al. identified retinoic acid-associated orphan receptor-alpha (RORA) in 2010, which is associated with autism [18]. The expression of this gene has been found to decrease in the lymphoblastoid cell line and post-mortem brain tissue of individuals with autism spectrum disorder. Sarachana and colleagues have also found that androgens (especially dihydrotestoste-rone) and estrogens (beta-estradiol) suppress RORA expression [9]. The *RORA* belongs to a gene family related to the retinoid acid receptor. This gene may play a role in many different pathways that affect the reproductive performance of animals. Previous studies have shown that some mutations of this gene can cause fertility problems in sheep. So, the *RORA* has a significant impact on fertility in animals. There has been some recent research on this topic. These studies show that it is important for sheep breeders to monitor mutations of the *RORA* gene to increase the fertility of sheep [19]. In this way, a healthier and more fertile sheep population can be achieved [19]. Genetic and functional analyses have found that *RORA* has regulatory effects on over 2500 genes, and more than 400 of these genes are considered risky for autism [20]. These studies suggest that *RORA* has a significant role in the reproductive system. 

To investigate the potential use of DNA markers in improving sheep fertility, a study was conducted to examine the link between the identification of *RORA* gene polymorphisms and twin births in a population of 106 Akkaraman sheep. Our study is the first study in Akkaraman sheep breed, and when the literature is examined, it is seen that our study is an original study and there is not enough data regarding it.

In putative intron 1, a mutation site consisting of 23 base pairs with three genotypes (II, ID and DD) was identified in this region. It can be hypothesized that the combination of the ID genotype and the II genotype leads to the production of homozygous DD genotypes, which are associated with larger litter sizes and increased fertility. From this point of view, we can conclude that the 23-bp index of the *RORA* gene has an effect on the pregnancy of Akkaraman ewes. When the literature was reviewed, twinning rate and *RORA* gene polymorphism were studied in 532 female Australian White ewes and as a result of this study, it was concluded that 23-bp index variants can be evaluated as molecular markers for second and third born litter size of ewes [19]. Microsatellite loci were studied to evaluate the linkages between various Akkaraman sheep breeds (Karakaş, Kangal and Savak) and other sheep breeds (Morkaraman, Awassi and Norduz) living in the same region. A total of 594 animals were analyzed and the results showed that Akkaraman breeds (Karakaş, Kangal and Savak) can be distinguished from each other based on microsatellite loci data. The findings of this study provide valuable information about the genetic structure of these sheep breeds and their relationships with each other. The study results suggest that the different genotypes observed in Savak- Akkaraman sheep may be attributed to their different breeding histories, geographical distribution, ecological conditions or selective pressures [21].

Calpastatin (*CAST*) and Growth Differentiation Factor 9 (*GDF9*) gene polymorphisms were investigated in 50 blood samples from Akkaraman sheep breed. The results revealed that Akkaraman sheep breed carries the GDF9-G1 mutation and shows sufficient genetic diversity in terms of *CAST* gene [22]. 

The impact of CAST-MspI, DGAT1-AluI, and IGF-1-Bsp143II polymorphisms on body weights was examined in Akkaraman lambs. The study revealed that the TC genotype had a beneficial effect on birth weight in the Akkaraman sheep breed [2].

The polymorphic structures of Myostatin (*MSTN*) gene locus of local Morkaraman sheep were analyzed and the distribution of genotype and allele frequencies were determined. MSTN/HaeIII gene polymorphisms were analyzed using DNA samples from 262 Morkaraman sheep and the observed heterozygosity (Ho) value calculated for *MSTN* in the whole population was found to be significantly higher than the expected heterozygosity (He) value [23].

The findings of several studies suggest that genetic mutations may impact gene function by influencing gene expression and nuclear factors. Specifically, in a study focused on RORA, it was proposed that variations in the intron region of the *RORA* gene could affect RORA expression and consequently impact *RORA*-controlled genes such as *CYP19A1* and *HSD17B10 *[20]. As a result of this effect, it is suggested that estrogen production may be regulated, follicular development may be impacted, and ultimately, reproduction may also be affected.

## Conflict of Interest:

There is no conflict of interest between the authors.
